# Identification and characterization of a galacturonic acid transporter from *Neurospora crassa* and its application for *Saccharomyces cerevisiae* fermentation processes

**DOI:** 10.1186/1754-6834-7-20

**Published:** 2014-02-06

**Authors:** J Philipp Benz, Ryan J Protzko, Jonas MS Andrich, Stefan Bauer, John E Dueber, Chris R Somerville

**Affiliations:** 1 Energy Biosciences Institute, University of California Berkeley, Berkeley, CA, USA; 2 Department of Molecular and Cell Biology, University of California Berkeley, Berkeley, CA, USA; 3 Department of Bioengineering, University of California Berkeley, Berkeley, CA, USA; 4 Department of Plant and Microbial Biology, University of California Berkeley, Berkeley, CA, USA; 5 present address: Institute of Environmental and Sustainable Chemistry, Technische Universität Braunschweig, Braunschweig, Germany

**Keywords:** Pectin, D-galacturonic acid, *Neurospora crassa*, Sugar transport, *Saccharomyces cerevisiae*, Metabolic engineering, Bioconversion, *Meso*-galactaric acid, L-galactonic acid

## Abstract

**Background:**

Pectin-rich agricultural wastes potentially represent favorable feedstocks for the sustainable production of alternative energy and bio-products. Their efficient utilization requires the conversion of all major constituent sugars. The current inability of the popular fermentation host *Saccharomyces cerevisiae* to metabolize the major pectic monosaccharide D-galacturonic acid (D-GalA) significantly hampers these efforts. While it has been reasoned that the optimization of cellular D-GalA uptake will be critical for the engineering of D-GalA utilization in yeast, no dedicated eukaryotic transport protein has been biochemically described. Here we report for the first time such a eukaryotic D-GalA transporter and characterize its functionality in *S. cerevisiae*.

**Results:**

We identified and characterized the D-GalA transporter GAT-1 out of a group of candidate genes obtained from co-expression analysis in *N. crassa*. The *N. crassa* Δ*gat-1* deletion strain is substantially affected in growth on pectic substrates, unable to take up D-GalA, and impaired in D-GalA-mediated signaling events. Moreover, expression of a *gat-1* construct in yeast conferred the ability for strong high-affinity D-GalA accumulation rates, providing evidence for GAT-1 being a *bona fide* D-GalA transport protein. By recombinantly co-expressing D-galacturonate reductase or uronate dehydrogenase in yeast we furthermore demonstrated a transporter-dependent conversion of D-GalA towards more reduced (L-galactonate) or oxidized (*meso*-galactaric acid) downstream products, respectively, over a broad concentration range.

**Conclusions:**

By utilizing the novel D-GalA transporter GAT-1 in *S. cerevisiae* we successfully generated a transporter-dependent uptake and catalysis system for D-GalA into two products with high potential for utilization as platform chemicals. Our data thereby provide a considerable first step towards a more complete utilization of biomass for biofuel and value-added chemicals production.

## Background

The development of sustainable energy and chemical production methods are among the greatest societal challenges facing our generation. Biofuel and green chemistry efforts utilizing renewable lignocellulosic plant biomass feedstocks contribute substantially towards these goals. However, to maximize economic viability, these processes must implement the complete conversion of all major constituent feedstock sugars. Pectin represents a main plant cell-wall polysaccharide that, unlike cellulose and hemicellulose, is not commonly utilized, especially in *Saccharomyces cerevisiae* (*S. cerevisiae*), for biofuel and platform chemical production. Since pectin is most abundant in the primary cell walls of soft and growing tissues, agricultural waste streams from fruits and vegetables are particularly pectin-rich. Crops with a high pectin content of 20 to >40% include sugar beet pulp, citrus peels, and apple pomace [1-3], and the use of their respective waste streams for the production of biofuels has been attempted in several studies [4-8]. These residues are naturally largely devoid of lignin, already collected at the processing plant, and partly pretreated during the sugar and juice extraction procedure, warranting their high potential as biofuel feedstocks [9,10]. The pectic cell wall fraction can be inexpensively and efficiently hydrolyzed into its component monosaccharides by enzymatic processes, as it is considerably less recalcitrant than other plant polysaccharides, such as cellulose. Despite these advantages, pectin-rich sugar beet pulp and citrus peels are currently disposed of in landfills or dried for use as a low-value cattle feed, an expensive procedure that significantly reduces profit margins. Utilizing these waste streams would augment current biofuel production efforts without creating direct or indirect land-use changes.

Pectin comprises a heterogeneous polysaccharide family with four main structural classes: homogalacturonan (HG), rhamnogalacturonan I (RG-I), and the substituted HGs rhamnogalacturonan II (RG-II) and xylogalacturonan (XG) (for review, see [11,12]). All pectic structures have α-D-galacturonic acid (D-GalA) as a major backbone component linked at the *O*-1 and the *O*-4 positions. This monosaccharide alone can comprise up to 70% of pectin; the remaining sugars include L-arabinose (L-Ara), D-galactose (D-Gal), L-rhamnose (L-Rha), and D-xylose (D-Xyl).

Engineering an organism to utilize D-GalA promises an attractive route for converting a currently underutilized, relatively abundant sugar into a biofuel or platform chemical. The yeast *S. cerevisiae* is currently the most attractive production host and remains the most popular microorganism for industrial fermentation strategies to produce bioethanol. Its advantages include a high tolerance to growth inhibitors from lignocellulose hydrolysates as well as ethanol, the ability to withstand low pH conditions that eradicate many bacterial contaminants, fast fermentation kinetics, and the suitability for many rounds of recycling [13,14]. Ample engineering efforts have already been undertaken to utilize glucose, xylose, and arabinose. Unfortunately, *S. cerevisiae* cannot metabolize D-GalA, because it lacks the genes encoding a catabolic pathway [15-17]. When fermenting hydrolysates from pectin-rich feedstocks, this could, therefore, lead to the accumulation of D-GalA in the broth, which was shown to be inhibitory to the fermentation of D-Gal, L-Ara, and D-Xyl [18]. A possible approach to overcome this problem is metabolic engineering. In this case, the genes encoding the necessary enzymes for D-GalA metabolism derived from organisms capable of utilizing this sugar could be heterologously expressed in yeast. Such pathways have been described in bacteria, such as *Escherichia coli* and *Erwinia*[19-22], as well as more recently in filamentous fungi, such as *Aspergillus niger, Trichoderma reesei* (anamorph of *Hypocrea jecorina*) and *Botrytis cinerea*[23-28] (also see [29] for further review).

A recent attempt to engineer *S. cerevisiae* strains carrying a bacterial D-GalA catabolic pathway met with considerable challenges in expressing functional enzymes [16,30]. Moreover, even though D-GalA was demonstrated to be able to enter *S. cerevisiae* cells under certain conditions (through an as-yet unidentified, low-affinity and channel-like pore at acidic pH close to its pK_a_ of about 3.5 [31]), it was reasoned that the optimization of D-GalA transport will be essential for the successful engineering of D-GalA utilization in yeast [14,16]. Although prokaryotic D-GalA transport systems are well known [32-35], these are notoriously difficult to express functionally in a eukaryotic host. However, so far no such transport protein has been described in a Eukaryote. Here we report such a eukaryotic D-GalA transporter. The corresponding gene was identified through a transcriptomics analysis of pectin degradation by the model filamentous fungus *Neurospora crassa* (*N. crassa*) [36], followed by a genetic analysis and the biochemical characterization of the encoded protein. Moreover, we show the transporter to be functional when heterologously expressed in *S. cerevisiae* and useful for the conversion of D-GalA to downstream products. Our findings are therefore an important step towards the effective utilization of pectin-rich feedstocks for the production of platform chemicals or biofuels.

## Results

### Identification of NCU00988 from *N. crassa* as a candidate D-galacturonic acid transporter

**Figure 1:**
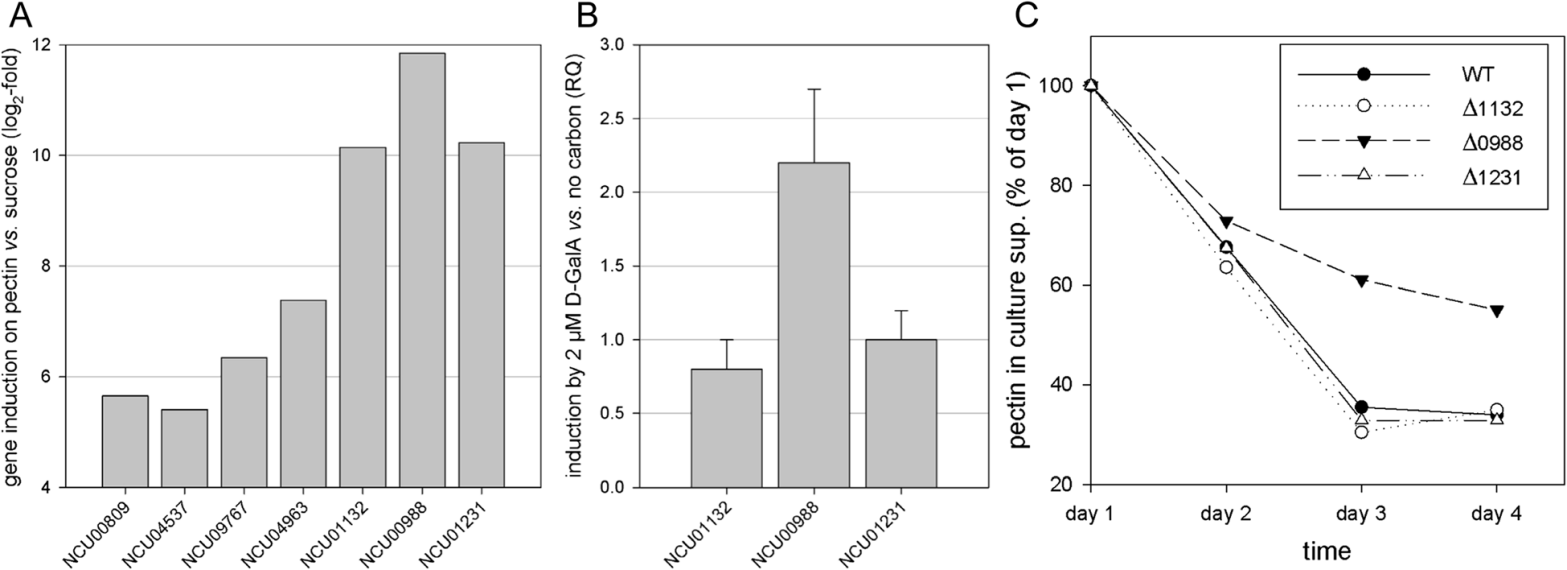
**Identification of NCU00988 as a candidate D-galacturonic acid (D-GalA) transporter. (A)** Induction of selected transporter genes after transfer for 4 h to pectin versus sucrose. The log_2_-fold induction of seven major facilitator superfamily (MFS)-type transporters from the most pectin-specific co-expression cluster as determined by Cuffdiff [37] is shown. Data were calculated from independent triplicate cultures. **(B)** Relative expression levels of the top three pectin-induced transporter genes in *N. crassa* as determined by quantitative PCR. Sucrose pre-grown mycelia were transferred to Vogel’s medium with 2 μM D-GalA or w/o carbon source (no carbon). Samples were taken 4 h after transfer. Relative transcript quantities (RQ) are depicted where 1 represents the transcription level on medium w/o carbon source. Only NCU00988 showed a response to the presence of D-GalA. Bars represent standard deviations (n = 3). **(C)** Pectin consumption phenotype. The deletion strains of the top three pectin-responsive transporters were grown for 4 days on 1% pectin and the consumption of pectin followed over time by measuring the remaining substrate concentration in the culture supernatants by the phenol-sulfuric acid method. Data represent the mean of triplicates and are plotted relative to the values at day 1 (as 100%). Only the deletion strain for NCU00988 (black triangle) displayed a strong delay in pectin consumption. WT, wild-type.

To identify candidate D-GalA transporters, we took advantage of a recently generated polysaccharide-biased co-expression matrix [37]. In that study, the whole-genome expression pattern of *N. crassa* cultures 4 h after transfer to cellulose, xylan, pectin, orange peel powder, sucrose, or no carbon were hierarchically clustered. Analysis of these transcriptomic data revealed groups of genes that specifically responded to the presence of the three main plant cell-wall polysaccharides - cellulose, hemicellulose, and pectin. Of approximately 160 genes encoding major facilitator superfamily (MFS)-type transporters in the *N. crassa* genome, only nine were found in the most pectin-specific cluster (10.1). Two of these, the allantoate permease (NCU02653) and the pantothenate transporter gene *liz1*, were considered to be less likely candidates due to their homology to non-sugar transporters and were omitted from further analyses. The induction by pectin versus sucrose (as a broadly repressing control condition) for the other seven candidates is depicted in Figure 1A. The three transporter genes with the strongest induction by pectin (>1,000-fold; log_2_ >10) were NCU01132, NCU00988, and NCU01231. For these we tested whether they are specifically induced by D-GalA alone. To this end, cultures of *N. crassa* wild-type (WT) pre-grown in sucrose for 16 h were transferred to either no carbon (de-repression but no induction) or very low concentrations of D-GalA (2 μM) and incubated for another 4 h. Under these conditions we expected a highly specific response. As shown in Figure 1B, only the NCU00988 gene displayed a significant induction >2-fold over the starvation condition without added sugar, whereas NCU01132 and NCU01231 did not respond at all. Moreover, when grown on Vogel’s medium with 1% citrus peel pectin as the sole carbon source, only the deletion strain for NCU00988 (Δ0988) exhibited a marked delay in pectin consumption over time (Figure 1C). Taken together, these data demonstrate that NCU00988 is of major importance for pectin utilization in *N. crassa*, potentially through the transport of the backbone monosaccharide D-GalA. We therefore decided to rename NCU00988 as the most promising candidate gene *gat-1*, for *g*alacturonic *a*cid *t*ransporter-1.

### The *N. crassa* deletion strain of NCU00988 (Δ*gat-1*) is impaired in growth on pectins and unable to take up D-galacturonic acid

**Figure 2:**
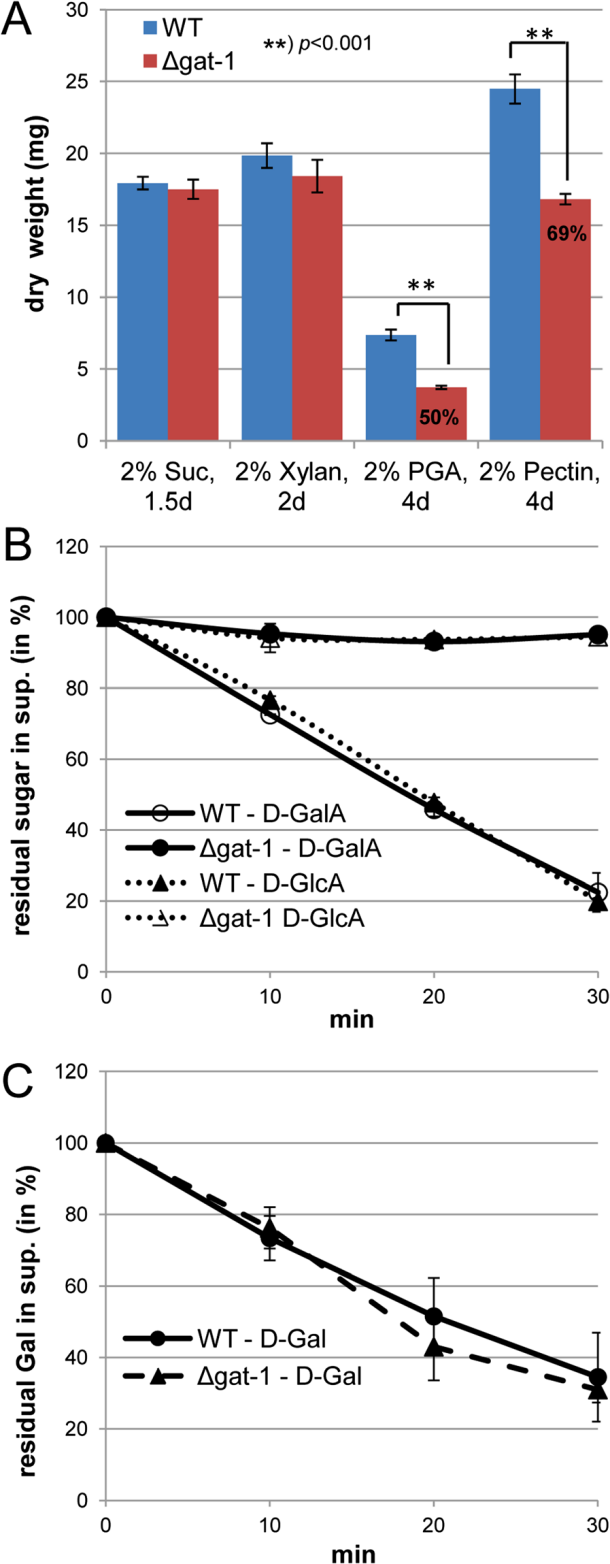
**The NCU00988 deletion strain Δ*gat-1 *is unable to take up D-galacturonic acid (D-GalA). (A)** Carbon source-specific growth phenotype. Conidia of *N. crassa* wild-type (WT) and the ∆*gat-1* deletion strain were inoculated into 3-mL cultures containing various 2% carbon sources and incubated at 25°C, 250 rpm in the light. At the indicated times (1.5 to 4.0 days) the mycelia were harvested and the dry weight determined. Bars represent standard deviations (n = 5). The ∆*gat-1* strain displayed a specific growth reduction on pectic substrates (polygalacturonic acid (PGA) and pectin from citrus peel). The extent of the biomass reduction is indicated (in % of WT). **(B,C)** Monosaccharide transport assays. Sucrose pre-grown *N. crassa* WT and ∆*gat-1* mycelia were transferred for 4 h to 0.5% pectin to induce the pectinolytic response and subsequently to the reaction solution containing 90 μM each of the indicated monosaccharides and Vogel’s salts (at pH 5.8). The cultures were incubated in the reaction solution for 30 minutes at 25°C, 250 rpm in the light. Aliquots of the supernatant were taken at regular intervals and the remaining sugar concentrations analyzed by HPAEC-PAD. Bars represent standard deviations (n = 3). ∆*gat-1* was found to be unable to transport D-GalA as well as D-glucuronic acid (D-GlcA). Suc, sucrose.

To test whether the loss of GAT-1 leads to a broad or specific phenotype, the corresponding deletion strain (Δ*gat-1*) was grown alongside the *N. crassa* WT on sucrose, xylan, and two pectic carbon sources (polygalacturonic acid (PGA) and citrus peel pectin) and the mycelial dry weight was determined after 1.5, 2.0, or 4.0 days, respectively, depending on the carbon source (Figure 2A). Under these conditions, the amount of accumulated biomass was found to be significantly reduced only on the pectic substrates, corroborating our earlier findings.

We next set out to test whether the growth inhibition on pectin could be directly linked to loss of D-GalA transport activity of the deletion strain. For this, sucrose pre-grown cultures of WT and Δ*gat-1* were first transferred for an additional 4 h to Vogel’s solution containing 0.5% pectin as the sole carbon source to induce the pectic response, before being transferred to the reaction solution containing 90 μM of either D-Gal, D-GalA, or glucuronic acid (D-GlcA). The residual concentrations of the monosaccharides in the reaction supernatant were measured by high pH-anion exchange-chromatography with pulsed amperometric detection (HPAEC-PAD) and plotted over time in Figures 2B and C. Whereas the transport speed for D-Gal was found to be WT-like in the Δ*gat-1* strain (Figure 2C), the transport of both D-GalA and D-GlcA was almost completely abolished (Figure 2B). These findings provide evidence that the physiological function of GAT-1 is indeed the transport of D-GalA (and the minor component D-GlcA) during fungal growth on pectin.

### GAT-1 is required for D-GalA-mediated signaling processes in *N. crassa*

**Figure 3:**
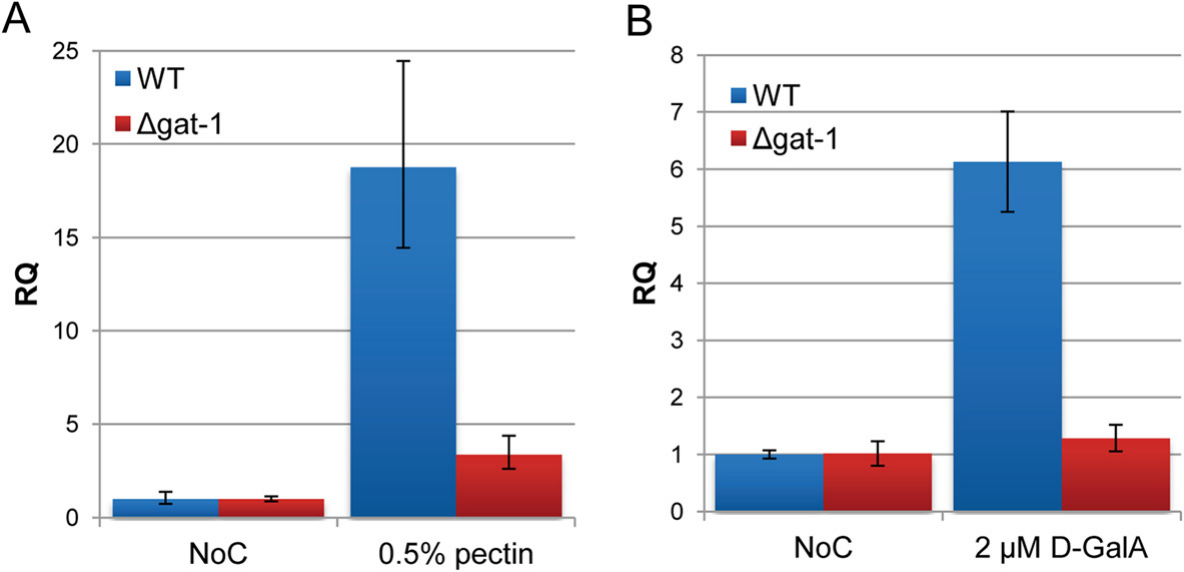
**GAT-1 is required for D-galacturonic acid (D-GalA)-mediated induction of pectinases. (A,B)** Relative expression levels of the exo-polygalacturonase gene *gh28-2* (NCU06961) in *N. crassa* as determined by quantitative PCR. Sucrose pre-grown WT and ∆*gat-1* mycelia were transferred to medium with 0.5% citrus peel pectin, 2 μM D-GalA or w/o carbon source (NoC). Samples were taken 4 h after transfer. Relative transcript quantities (RQ) are depicted, where 1 represents the transcription level on NoC. The ∆*gat-1* deletion strain displayed a strong reduction of pectinase induction in both conditions. WT, wild-type.

The near-complete loss of all D-GalA transport activity in the Δ*gat-1* deletion strain suggests that GAT-1 likely represents the only access route for D-GalA in *N. crassa*, at least under pectin-induced conditions. However, RNAseq data obtained after transfer of mycelia to either sucrose, no carbon, or pectin (see Additional file 1: Figure S1) show that *gat-1* is not only strongly induced by pectin but also considerably de-repressed under starvation conditions, and therefore probably part of the carbon scouting machinery [38]. To assess whether the absence of GAT-1 would also have an impact on pectin or, more specifically, D-GalA-mediated signaling events, we analyzed the induction of one of the major polygalacturonase genes (*gh28-2*) [37] in Δ*gat-1* cultures versus the WT. For this purpose, sucrose pre-grown mycelia were transferred to either 0.5% pectin or 2 μM D-GalA for 4 h before harvesting of RNA and measuring the transcript abundance of *gh28-2* by quantitative qPCR (Figure 3). The induction of *gh28-2* in the Δ*gat-1* strain was found to be strongly reduced on pectin (<20% of WT; Figure 3A) and completely undetectable on D-GalA only (Figure 3B). These data corroborate that GAT-1 is required for the uptake of D-GalA and show that it is furthermore an integral part of the fungal pectin signaling pathway.

### The transport of D-galacturonic acid and quinic acid are separate physiological processes

The (generic) annotation of the *N. crassa* genome by the Broad Institute classifies the *gat-1* gene (NCU00988) as “MFS quinate transporter”, which presumably can be attributed to the fact that GAT-1 and the quinate permease (NCU06026; *quinate-Y*[39-41]; Broad Institute of Harvard and MIT [http://www.broadinstitute.org/]) indeed share considerable homology (39% identity, 54% similarity [42]; Additional file 2: Figure S2). Intriguingly, D-GalA and quinate are also similar in their structure and conformation: quinic acid (*qa*) being a cyclic polyol, with the carboxyl and at least one hydroxyl substituent in identical equatorial conformation and ring position when presented in six-membered ring chair form structures as shown in Additional file 3: Figure S3A. To test whether the two transporters are also functionally redundant, we performed transport assays with *N. crassa* WT mycelia that had been pre-grown in sucrose and were then transferred for 4 h to Vogel’s medium containing both 0.5% pectin and 100 μM *qa* for the parallel induction of both uptake systems. When the uptake of both substrates was measured over the course of 40 minutes, the absence of GAT-1 did not affect *qa* uptake rates (Additional file 3: Figure S3B), just as the absence of the quinate permease did not negatively affect the uptake of D-GalA (but rather led to a slightly accelerated D-GalA consumption; Additional file 3: Figure S3C). In contrast, the deletion strain for the quinate permease (Δ6026) was almost completely unable to take up *qa*, indicating that it is the only physiologically relevant transporter for this compound. Therefore, despite their homology, our data suggest a distinct, not overlapping role for GAT-1 and the quinate permease in *N. crassa*.

### Heterologous expression of GAT-1 in *S. cerevisiae* confers the ability to take up D-galacturonic acid with high affinity

**Figure 4:**
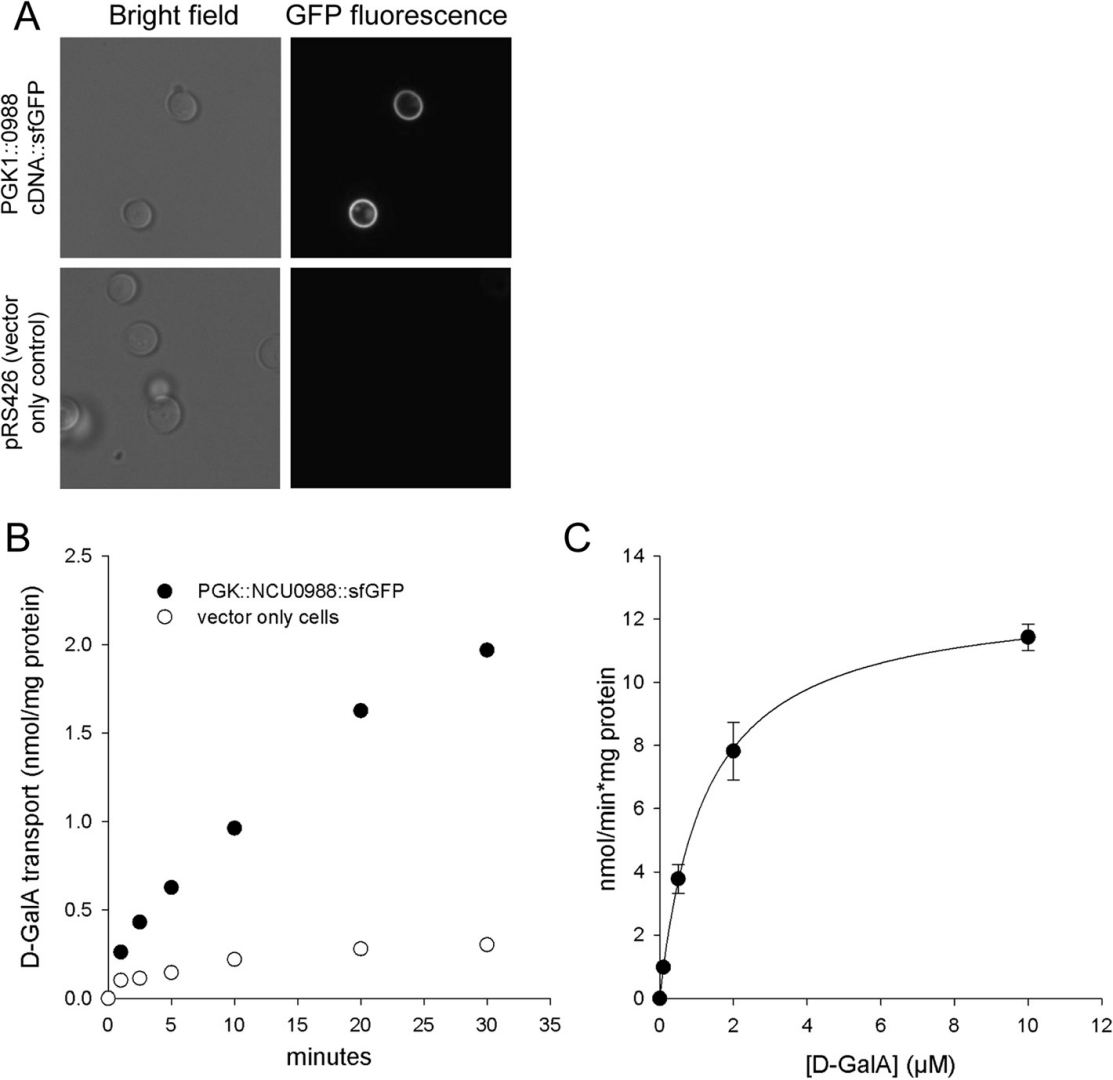
**Heterologous expression of *gat-1 *in yeast confers the ability to uptake D-galacturonic acid (D-GalA) with high affinity.** The cDNA of *gat-1* was fused to the super folder-green fluorescent protein (sfGFP) under the control of the phosphoglycerate kinase 1 (PGK1) promoter and transformed into yeast (*S. cerevisiae* strain BY4742). **(A)** The incorporation of the construct into the plasma membrane was followed by confocal microscopy (100 × oil; upper panels), whereas no GFP fluorescence could be observed in the vector-only transformed control cells (lower panels). **(B)** D-GalA transport by the *S. cerevisiae* strains as described above. Shown is D-GalA transport by yeast with (closed circles) or without (open circles) GAT-1. The initial concentration of D-GalA was 50 μM. All values are the mean of two measurements. **(C)** Kinetics of D-GalA transport by GAT-1. The transport rate was determined as a function of D-GalA concentration (ranging from 0.1 μM to 10 μM) by yeast strains expressing *gat-1-sfGFP* and was normalized by total protein concentration.

As our goal was to find a D-GalA transporter that would be useful for biotechnological applications in yeast, we transformed *S. cerevisiae* with a construct harboring the *gat-1* cDNA fused C-terminally to green fluorescent protein (GFP) and driven by the strong phosphoglycerate kinase 1 (PGK1) promoter. Confocal microscopy confirmed correct targeting of the construct into the plasma membrane (Figure 4A). When the yeast cells were subjected to D-GalA uptake assays using (^3^H)-D-GalA as radio-labeled tracer, we observed substantial D-GalA import over the background of cells transformed with the empty vector (Figure 4B), indicating that GAT-1 was active in the heterologous system. In kinetic experiments with varying D-GalA concentrations, we subsequently determined GAT-1 to be a high-affinity transporter, with a Michaelis constant (K_m_) value of 1.2 +/- 0.1 μM (mean +/- SD) and a maximum velocity (V_max_) of 12.8 +/- 0.4 nmol minute^-1^ mg protein^-1^ (Figure 4C). The additional observation that D-GalA transport in *N. crassa* is sensitive to the presence of very low concentrations (30 μM) of the well-known uncoupling reagent carbonylcyanide *m*-chlorophenylhydrazone (CCCP; Additional file 3: Figure S3D) suggests that GAT-1, which is clearly responsible for the vast majority of D-GalA import under these conditions, utilizes the proton gradient across the plasma membrane for its function, and therefore likely acts as a H^+^/D-GalA symporter.

### Engineering of *S. cerevisiae* for the production of L-galactonic acid and *meso*-galactaric acid from D-galacturonic acid

**Figure 5:**
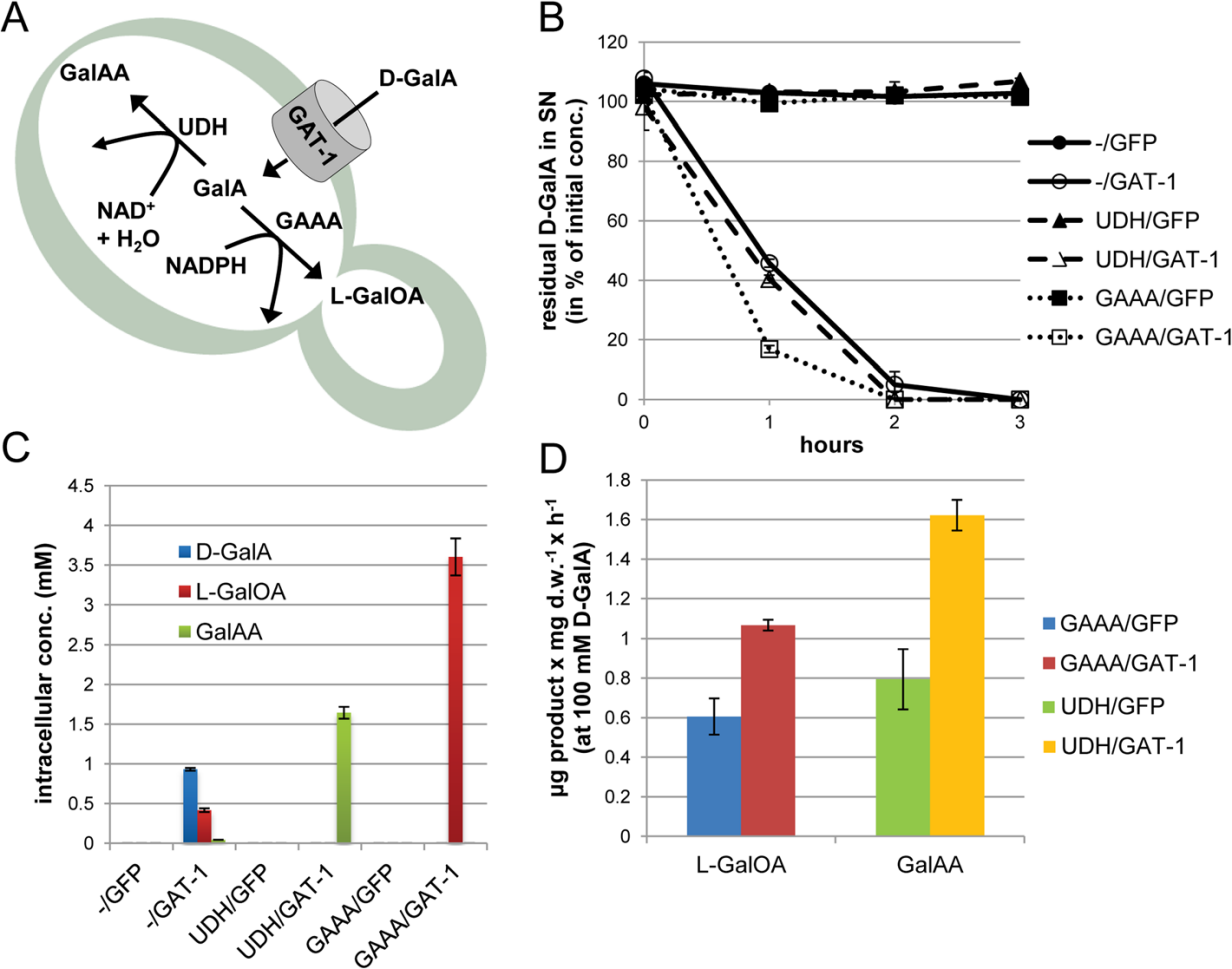
**Bioconversion of D-galacturonic acid (D-GalA) to downstream products by genetically engineered yeast strains. (A)** D-GalA can be converted to *meso*-galactaric acid (GalAA) and L-galactonate (L-GalOA) in *S. cerevisiae* strains heterologously expressing uronate dehydrogenase (UDH) or D-galacturonic acid reductase (GAAA), respectively, using endogenous cofactors. **(B)** Bioconversion yeast strains expressing GAT-1 exhibit rapid, high-affinity uptake of D-GalA (at pH 5.8 and an initial D-GalA concentration of 90 μM). **(C)** Intracellular products were detected by liquid chromatography coupled to tandem mass-spectrometry of chloroform:methanol:water-extracted yeast cells from the 1-h time point samples and their accumulation was found to be transporter-dependent. **(D)** Even at high D-GalA conditions (100 mM, pH 6.0) co-expression of GAT-1 in GAAA- or UDH-expressing yeast strains increases bioconversion product accumulation by an average 1.8- and 2.1-fold, respectively. NAD^+^, Nicotinamide adenine dinucleotide; NADPH, Nicotinamide adenine dinucleotide phosphate (reduced); GFP, green fluorescent protein.

To test the utility of GAT-1 in D-GalA bioconversions using *S. cerevisiae* as a production host, we built a strain with *gat-1-GFP* integrated into the chromosome as well as a strain expressing only GFP from the same chromosomal location, as a control for non-specific uronic sugar transport. These base strains were then transformed to express either *Aspergillus niger* D-galacturonate reductase (GAAA) or *Agrobacterium tumefaciens* uronate dehydrogenase (UDH) [26,43], which reduce D-GalA to L-galactonic acid (L-GalOA) or oxidize it to *meso*-galactaric acid (GalAA), respectively (Figure 5A). We observed rapid uptake in strains expressing the transporter, whereas the D-GalA concentration in the samples of the GFP control strains remained constant during the 3-h assay at high-affinity concentrations (90 μM) and pH 5.8 (Figure 5B). The intracellular production of L-GalOA and GalAA, as determined by liquid chromatography coupled to tandem mass-spectrometry (LC-MS/MS) from chloroform:methanol:water-extracted cells, demonstrated production of the respective downstream products in a GAT-1-dependent manner (Figure 5C). Interestingly, whereas GalAA production was enzyme (UDH)dependent, a basal level of incomplete D-GalA bioconversion to L-GalOA was observed without expression of GAAA (strain -/GAT-1). However, expression of the reductase resulted in a higher product yield (GAAA/GAT-1).

Passive uptake of D-GalA by WT yeast has been observed at high concentrations of this substrate (50 to 200 mM), which is presumably within the concentration range found in hydrolysates from pectin-rich feedstocks. This uptake phenomenon was shown to be largely dependent on the concentration of D-GalA and pH [31]. The expression of a primary or secondary active transporter could plausibly improve D-GalA transport rates even at high concentrations and/or sustain import as extracellular concentrations decrease. To test whether heterologous expression of the high-affinity transporter GAT-1 will also have beneficial effects in high-D-GalA media conditions we measured L-GalOA and GalAA production in our bioconversion strains at 100 mM D-GalA and pH 6.0. After 1 h incubation, we observed an average 1.8-fold improvement in L-GalOA and an average 2.1-fold improvement in GalAA production with expression of GAT-1-GFP over GFP alone (Figure 5D).

## Discussion

In the present study, we identified and characterized the first D-GalA transporter from a eukaryotic organism out of a group of candidate genes derived from a co-expression analysis performed in *N. crassa*. Genetic and biochemical analyses show that the *N. crassa* Δ*gat-1* deletion strain is substantially affected in growth on pectic substrates and is unable to take up D-GalA from the medium. Moreover, expression of a *gat-1* construct in yeast conferred the ability for robust intracellular D-GalA accumulation rates, providing evidence for GAT-1 being a *bona fide* D-GalA transport protein. In view of the fact that the Δ*gat-1* deletion strain is also impaired in D-GalA-mediated signaling events, this furthermore corroborates the notion of an activation system for pectinolytic genes responding to D-GalA ([25] and references therein) and strongly suggests that GAT-1 is an integral part of the D-GalA signaling system in *N. crassa* - either by taking up D-GalA as the signaling molecule (or a precurser thereof) or by acting as a receptor itself.

Several bacterial D-GalA transport systems have already been described, such as ExuT, TogT, TogMNAB and KdgM from *E. coli* and *Erwinia*, a bacterial soft-rot causing plant pathogen. KdgM (a porin of the outer membrane [32]), TogMNAB (an ABC superfamily member in the inner membrane [33]) and TogT (an MFS-type transporter of the glycoside-pentoside-hexuronide (GPH) sub-family also located in the inner membrane [34]) are responsible for D-GalA oligosaccharide transport, whereas ExuT was shown to exclusively transport monomeric D-GalA [35]. Functionally, therefore, GAT-1 seems most similar to ExuT. Indeed, both are high-affinity transporters (K_M_ of 25 μM for ExuT versus 1.2 μM for GAT-1), display uptake rates in a very similar range (V_max_ of 38 nmol min^-1^ mg protein^-1^ for ExuT versus 12.8 nmol min^-1^ mg protein^-1^ for GAT-1), transport D-GlcA in addition to D-GalA and are sensitive to the presence of uncouplers [35]. However, it is noteworthy to mention that the kinetic parameters of GAT-1 were recorded as super-folder (sf)GFP fusion protein heterologously expressed in *S. cerevisiae*, and that therefore the behavior of the native protein in *N. crassa* might differ from these values. Despite belonging to the same MFS-type superfamily, ExuT (345 aa in *E. chrysanthemi*) and GAT-1 (537 aa in *N. crassa*) do not share any recognizable protein or sequence similarity and, therefore, have to be considered functional analogs.

Orthologs of GAT-1 (as well as the quinate permease) are found throughout species of Ascomycota and Basidiomycota (see Additional file 2: Figure S2), with the notable exception of *Tuber melanosporum*, which only seems to have a quinate permease and no additional homolog of GAT-1, and *S. cerevisiae*, which does not have a clear ortholog of either. Our phylogenetic analysis shows that GAT-1 homologs can be clustered into three clades, each with representatives from both Ascomycota and Basidiomycota (Additional file 2: Figure S2). It furthermore suggests that the GAT-1-containing clade 1 and the quinate permease clade both share a common ancestor and are equally derived from clade 2 and the more distantly related clade 3. While this manuscript was in proof, Enquist-Newman *et al*. (2014) discovered another uronic acid transporter (DHT1 from *Asteromyces cruciatus*) for the uptake of the alginate monomer 4-deoxy-L-erythro-5-hexoseulose uronate (DEHU), which represents a homolog to GAT-1 and the quinic acid permeases and is another good example of the fact that these two transporter families are evolutionarily well related [44].

Considering the number of GAT-1 homologs per genome, *Botrytis cinerea* seems most diverse, having GAT-1 homologs in all three clades, whereas others, such as *Aspergillus nidulans* and *Trichoderma reesei*, have one GAT-1 ortholog (in clade 1) and one or two paralogs in clade 2, respectively. Interestingly, *Aspergillus niger* only has a clade-2 homolog to GAT-1. However, since the corresponding gene (An14g04280) has been previously shown to be co-regulated with genes involved in pectin degradation and D-GalA catabolism and failed to express in absence of *gaaB*, the hypothesis of it being a D-GalA transporter has been brought forward [25,45]. Additionally, Zhang *et al*. (2013) recently reported the An14g04280 homolog in *Botrytis cinerea* (*BcHxt15*) to be specifically induced by D-GalA and the corresponding mutant to be impaired in growth on D-GalA, which led them to suggest that BcHXT15 contributes to D-GalA uptake [46]. Even though no representative has been properly biochemically characterized yet, considering these lines of evidence, it seems possible that the clade-2 homologs are also *bona fide* D-GalA transport proteins.

Regarding biotechnological applicability, we demonstrated a transporter-dependent conversion of D-GalA into two different molecules, L-GalOA and GalAA, by recombinantly expressing D-galacturonate reductase (GAAA) and uronate dehydrogenase (UDH) in yeast, respectively. GalAA is an isomer of glucaric acid, which was identified as a “top value-added chemical” in a report by the Pacific Northwest National Laboratory and the National Renewable Energy Laboratory for its potential use as a building block for hydroxylated nylons [47-50]. Consequently, GalAA is also likely to be capable of producing desirable hydroxylated nylons. L-GalOA on the other hand is reported to have similar properties to the commodity compound D-gluconic acid and correspondingly holds potential as a chelator in a variety of industries (for example cosmetic, pharmaceutical, food) and can also be used as a chemical precursor to L-ascorbic acid [45,51,52]. Production of both of these chemicals was completely GAT-1-dependent at the low D-GalA concentrations typical for high-affinity transport (90 μM). Even at high D-GalA concentrations (100 mM/2%), at which non-specific uronic sugar uptake has been shown to be substantial [31], expression of the transporter increased product accumulation roughly 2-fold over that observed in control strains at pH 6.0. Thus, D-GalA utilization can be improved over a broad concentration range in engineered yeast strains expressing a heterologous transporter. Interestingly, whereas production of GalAA was dependent on both GAT-1 and UDH, production of L-GalOA did not require heterologous expression of GAAA. Therefore, a basal level of native D-GalA reductase activity is present in *S. cerevisiae*, though its origin remains unknown.

Utilization of D-GalA for the biosynthesis of biofuels, specifically ethanol, from pectin-rich feedstocks has been a focus of increasing attention in recent years. The isolation and demonstration of activity of this transporter in *S. cerevisiae* provides an important contribution towards realization of this goal. Although functional expression of the requisite pathway enzymes remains an unsolved challenge, an increasing number of alternative orthologous enzymes and pathways are currently being explored [53-55], which will potentially allow this issue to be overcome in the near future.

## Conclusions

In the present manuscript we have for the first time unambiguously identified and biochemically characterized a D-GalA transporter from a eukaryotic origin. GAT-1 was found to be the physiologically relevant D-GalA transporter in *N. crassa*, involved both in the uptake of D-GalA as a metabolite as well as for pectinase signaling events. Moreover, by heterologous expression of GAT-1 in combination with two downstream enzymes, we demonstrate successful transport and catalysis of D-GalA in the fermentation host *S. cerevisiae*, and thereby provide a considerable first step towards a more complete utilization of biomass for biofuel and value-added chemicals production.

### Strains, media, and growth conditions

*N. crassa* WT (FGSC #2489) and gene deletion strains used in this study were obtained from the Fungal Genetics Stock Center (FGSC; [http://www.fgsc.net]). *N. crassa* was grown on 1 × Vogel’s salts [56] with either sucrose (Fisher S3-12; Fisher Scientific, Chicago, IL, USA), or pectin (Sigma P9135; Sigma-Aldrich, St Louis, MO, USA), xylan (beechwood, Sigma X4252), orange peel powder (OPP [37]), or Avicel (PH 101, Fluka 11365) at the indicated concentrations (w/v) at 25°C and 200 rpm with constant light unless stated otherwise.

The yeast strain used in this study was BY4742 (MATα his3Δ1 leu2Δ0 lys2Δ0 ura3Δ0) [57]. Growth was usually performed in yeast extract peptone dextrose (YPD) media supplemented with 100 mg/L adenine hemisulfate and transformed strains were grown in the appropriate complete minimal dropout media, supplemented with 100 mg/L adenine hemisulfate. Yeast cultures were grown in 250-mL baffled flasks at 30°C and 250 rpm for microscopy and uptake experiments.

### Plasmids and cloning

For yeast expression purposes, the 2 μ plasmid pRS426, equipped with the PGK1 promoter and sfGFP as generated and described in [58], was used. The *gat-1* cDNA (with optimized Kozak sequence) was inserted between SpeI and ClaI sites using the primers AT**ACTAGT**AAAAATGGGTCTTTCGATAGGAAATAGG and AT**ATCGAT**AACATAAACCTCCACATGCTTCG (restriction sites in boldface). For genomic integration, the *gat-1* sequence (RefSeq: XM_958805.2) with a C-terminal GFP fusion, or GFP alone, with an upstream TDH3 promoter was flanked by respective 5′ and 3′ *ura3* homology regions using golden-gate cloning. The integration cassette was amplified by PCR and the resulting fragments were used in yeast transformations. The *Agrobacterium tumefacians udh* open reading frame [GenBank: BK006462.1] and a codon-optimized *Aspergillus niger GAAA* [GenBank: ABQ53587.1] were cloned into a *Cen6/leu2* vector derived from pRS316 [59] with a TDH3 promoter. For used gene sequences, see Table S1 in Additional file 4.

### Phenotypic analyses

Phenotypic analyses of deletion strains were usually performed in 24 deep-well plates in a volume of 3 mL over the course of 4 days. Dry weight was determined after an overnight incubation of the mycelial mass in aluminum pans in a 105°C oven. Pectin consumption over time was followed using the phenol-sulfuric acid assay (PSA) [60]. For this, pectin-grown *N. crassa* cultures were initially cleared by centrifugation at 20,000 × g for 5 minutes: 2.5 μL of the supernatant was diluted into 150 μL de-ionized (DI) water and mixed with 150 μL of a 5% phenol solution. Finally, 750 μL of concentrated sulfuric acid was quickly added, and the suspension was vortexed for 5 to 10 seconds. After about 7 minutes incubation at room temperature, 200 μL of the reactions were analyzed in a plate-reader spectrophotometer (Paradigm; Beckman-Coulter, Brea, CA, USA) at 487 nm.

### Phylogenetic analysis

Protein sequences of GAT-1, NCU06026 (quinate permease), and fungal homologs from representative ascomycete and basidiomycete species were retrieved from the NCBI database. Sequences were aligned and a phylogenetic tree constructed using the program Phylogeny.fr [61] (alignment by MUSCLE - excluding curation by Gblocks, phylogeny calculation by PhyML, and tree rendering by TreeDyn). Gal2p from *S. cerevisiae* was used as the out-group.

### Media shift assays for transcriptional studies

The standard *N. crassa* growth conditions for any experiments involving media switches were as follows: cultures were pre-grown from 9- to 10-day-old conidia for 16 h in 3 mL of 1 × Vogel’s salts plus 2% (w/v) sucrose in 24 deep-well plates. The mycelia were then washed three times in 1 × Vogel’s salts without added carbon (NoC) and transferred to 1 × Vogel’s salts plus the respective new carbon source (as indicated). The mycelial mass was harvested after an additional 4 h by quickly blotting dry on Whatman tissues and subsequent flash-freezing in liquid nitrogen to be stored at -80°C. Total RNA from frozen samples was isolated using zirconia/silica beads (0.5 mm diameter; Biospec, Bartlesville, OK, USA) and a Mini-Beadbeater-96 (Biospec) with 1 mL TRIzol reagent (Invitrogen/Life Technologies, Carlsbad, CA, USA) according to the manufacturer’s instructions. The total RNA was further digested with TURBO DNA-free (Ambion/Life Technologies) and purified using an RNeasy kit (Qiagen, Valencia, CA, USA). RNA concentration and integrity was checked by Nanodrop and agarose gel electrophoresis.

### Quantitative PCR

Quantitative RT-PCR was performed using the EXPRESS One-Step SYBR GreenER with Premixed ROX kit (Invitrogen/Life Technologies) and the StepOnePlus Real-Time PCR System (Applied Biosystems/Life Technologies). Reactions were performed in triplicate (each from three biological replicates) with a total reaction volume of 10 μL including 300 nM each of forward and reverse primers (Additional file 5: Table S2) and 75 ng template RNA. Data analysis was performed by the StepOne Software (Applied Biosystems) using the Relative Quantitation/Comparative CT (ΔΔCT) setting. Data were normalized to the endogenous control actin (NCU04173) with expression on sucrose or no carbon as the reference sample (as indicated).

### RNA-Seq

*N. crassa* cultures were pre-grown from 10-day-old conidia for 16 h in 100 mL of 1 × Vogel’s salts plus 2% (w/v) sucrose using 250-mL shake-flasks. The mycelia were then washed three times in 1 × Vogel’s salts without added carbon (NoC) and transferred to 2% sucrose or 1% (w/v) pectin for induction [37,62]. After an additional 4 h, the mycelial mass was harvested over a Whatman glass microfiber filter (GF/F) on a Buchner funnel and subsequently flash-frozen in liquid nitrogen to be stored at -80°C. RNA was then prepared as above.

Library preparation for RNA sequencing (RNA-Seq) was performed essentially as described by Benz *et al*. and Coradetti *et al*. [37,62]. These were sequenced in SR50 mode on an Illumina HiSeq2000 at the UC Davis Genome Center, and the files analyzed using the Illumina RTA 1.12 software. Mapping of the reads was done against the current version at the time of the *N. crassa* OR74A genome (v10) [39] using Tophat v1.2.0 (http://tophat.cbcb.umd.edu/) [63]. Transcript abundance was estimated with Cufflinks v0.9.3 in fragments per kilobase of transcript per million mapped reads (FPKMs) using upper quartile normalization and mapping against reference isoforms from the Broad Institute (http://cufflinks.cbcb.umd.edu/) [64-66]. Profiling data are available at the Gene Expression Omnibus (http://www.ncbi.nlm.nih.gov/geo/) [GEO: GSE42692]. Genes with a multiple-hypothesis-adjusted *P*-value of <0.05 using Cuffdiff were called as significantly differentially expressed between conditions. Independent triplicate cultures were harvested and analyzed for *N. crassa* WT on pectin and sucrose.

### Monosaccharide uptake assays

Transport assays were performed according to [58]. Briefly, *N. crassa* cultures were pre-grown from 9- to 10-day-old conidia for 16 h in 3 mL of 1 × Vogel’s salts plus 2% (w/v) sucrose using 24 deep-well plates. The mycelia were then washed three times in 1 × Vogel’s salts without added carbon (NoC) and transferred to 0.5% (w/v) pectin for induction. After an additional 4 h, the mycelia were washed again as above and transferred into the uptake buffer (1 × Vogel’s salts plus 90 μM monosaccharides or quinic acid, as indicated) for pre-equilibration and to reduce the dilution of the final uptake reaction. Two mycelia of the same genotype were combined into one well to increase the biomass at this stage. In the case of the addition of uncoupler, CCCP (in ethanol at the indicated concentrations; controls with ethanol only) was added at this stage and kept throughout the assay. After 5 minutes, the mycelia were transferred into fresh uptake buffer to start the reaction. Time points of the supernatants were usually taken at 0, 5, and up to 40 minutes. The samples were cleared by centrifugation (1 minute at 20,000 × g) and 50 μL of the supernatant diluted into 450 μL of DI water. The monosaccharide concentrations were then quantified by HPAEC-PAD (Thermo Fisher Scientific Inc., Bannockburn, IL, USA): for neutral sugars, a sample size of 25 μL was injected onto a Dionex CarboPac PA20 column (3 × 30 mm guard and 3 × 150 mm analytical) and eluted at 30°C using an isocratic mobile phase of 18 mM KOH at 0.4 mL/minute over 11 minutes. For uronic acids, 25 μL was injected onto a Dionex CarboPac PA200 column (3 × 30 mm guard and 3 × 250 mm analytical) and eluted at 30°C using a 50 mM to 170 mM sodium acetate gradient (in 0.1 M NaOH) over 8 minutes at 0.4 mL/minutes.

For mass spectrometry (MS) detection of quinic acid, samples were injected onto a Rezex RFQ Fast Fruit H^+^ (8%) (Phenomenex, Torrance, CA, USA) column (100 × 7.8 mm) and eluted at 55°C using an isocratic mobile phase of 0.5% formic acid at a flow rate of 0.3 mL/minute. An LTQ XL linear ion trap (Thermo Scientific) was used for MS and MS/MS detection in negative ion mode. The quinic acid precursor ion of m/z 191.2 was followed (isolation width m/z 2; CID setting 35; product scan range m/z 50 to 200), and for chromatogram processing the mass transition (MS/MS) of m/z 191.2 to m/z 127.2 was selected.

### Yeast bioconversion assays

Yeast strains were grown as described above and harvested at mid-log phase. Cells were washed twice in ½ × Vogel’s salts (pH 5.8) with 50 mM ethanol added as a non-fermentable carbon source and resuspended in a final volume to yield an optical density (OD)_600_ of 20. To each experimental condition, 0.5 mL of concentrated cells were added to 24-well deep-well blocks and 0.5 mL of 2 × solution was added to yield a final concentration of ½ × Vogel’s salts, 90 μM D-GalA (Fluka/Sigma-Aldrich 48280-F) and 50 mM ethanol at an OD_600_ of 10. Blocks were incubated at 30°C with 750 rpm of shaking until the appropriate time point. Samples were cleared by centrifugation and the supernatant was prepared and analyzed as described above. Cell pellets were washed 2 × in cold DI water and freeze-dried in pre-weighed tubes to calculate dry cell weight. For assaying intracellular metabolites, desiccated yeast pellets were subjected to bead-beating (1 minute) in a Mini-Beadbeater-96 (Biospec) with an added 2:2:1 (v/v/v) ratio of chloroform, methanol and water. The aqueous phase was collected after centrifugation at 14,000 rpm for 10 minutes and was subsequently concentrated into 60 μL of DI water after vacuum centrifugation. The resulting sample was used for MS and MS/MS detection as described above. The respective precursor ions and mass transitions followed were: D-GalA: m/z 193.1 to m/z 131.1, L-GalOA: m/z 195.1 to m/z 159.1; and GalAA: m/z 209.1 to m/z 85.1. For the calculation of intracellular metabolite concentrations, dry cell weight was converted to cell volume using the factor 2.2 μL/mg dry weight [31,67].

High-D-GalA concentration yeast bioconversions were performed in a similar manner as low-concentration uptake assays, but with some modifications. Cells were grown and harvested at mid-log and washed twice in 1 × PBS, pH 6.0, with an added 50 mM EtOH. The final cell suspensions in this buffer were adjusted to an OD_600_ of 20 and 0.5 mL of cells were added to each condition in a 24 deep-well block in triplicate. To this, 0.5 mL of solution was added to yield 100 mM D-GalA in 1 × PBS (pH 6.0) with an OD_600_ of 10. Blocks were incubated at 30°C with 750 rpm of shaking for 1 h. To assay for bioconversion products, a portion of the cell suspension was flash-frozen in liquid nitrogen and added as a 2:2:1 (v/v/v) ratio of chloroform, methanol and cell suspension. Washing the cell pellets was avoided to minimize product loss [31]. A duplicate portion of cell suspension of each condition was lyophilized to calculate dry cell weight. Aqueous phase extraction and product detection proceeded as described above.

### (^3^H)-Galacturonic acid transport assays in yeast

Transport assays were performed using a modified oil-stop method [58]. In short, yeast strains expressing *gat-1* fused to GFP were grown to an OD (600 nm) of 4.0 to 5.0 in selective media, washed 3 × with ice cold assay buffer (30 mM MES-NaOH (pH 5.6) and 50 mM ethanol), and resuspended to an OD of 40.0. To start transport reactions, 50 μL of cells were added to 50 μL of (^3^H)-D-GalA layered over 100 μL of silicone oil (Sigma 85419). Reactions were stopped by spinning cells through oil for 1 minute at 17,000 g, tubes were frozen in ethanol/dry ice, and tube-bottoms containing the cell pellets were clipped off into 1 mL of 0.5 M NaOH. The pellets were solubilized overnight, 5 mL of Ultima Gold scintillation fluid added, and counts per minute (CPM) determined in a Tri-Carb 2900TR scintillation counter. (^3^H)-D-GalA was purchased from ViTrax, Inc. (Placentia, CA, USA), and had a specific activity of 25 Ci/mmol and a purity of >99%. Kinetic parameters were determined by measuring the linear rate of (^3^H)-D-GalA uptake over 3 minutes for D-GalA concentrations between 0.1 and 10 μM. V_max_ and K_M_ values were determined by fitting a “Ligand binding”, “one site saturation” function to a plot of rates versus D-GalA concentrations by non-linear regression in SigmaPlot®. V_max_ values were normalized by total protein content using a Bradford assay. Kinetic parameters reported in the text are the mean ± SD from three separate experiments.

## Abbreviations

CCCP: carbonylcyanide m-chlorophenylhydrazone; D-Gal: D-galactose; D-GalA: D-galacturonic acid; GalAA: *meso*-galactaric acid (mucic acid); D-GlcA: D-glucuronic acid; DI: de-ionized; D-Xyl: D-xylose; GAAA: D-galacturonate reductase; GFP: green fluorescent protein; HG: homogalacturonan; HPAEC-PAD: high pH anion-exchange chromatography with pulsed amperometric detection; L-Ara: L-arabinose; LC-MS/MS: liquid chromatography coupled to tandem mass-spectrometry; L-GalOA: L-galactonate; L-Rha: L-rhamnose; MFS: major facilitator superfamily; MS: mass spectrometry; NAD+: Nicotinamide adenine dinucleotide; NADPH: Nicotinamide adenine dinucleotide phosphate (reduced); OD: optical density; PBS: phosphate-buffered saline; PGA: poly-galacturonic acid; PGK1: phosphoglycerate kinase 1; qa: quinic acid; qPCR: quantitative PCR; RG-I: rhamnogalacturonan I; RG-II: rhamnogalacturonan II; RQ: relative transcript quantities; RT-PCR: reverse transcriptase PCR; sfGFP: super-folder green fluorescent protein; suc: sucrose; UDH: uronate dehydrogenase; WT: wild-type; XG: xylogalacturonan; YPD: yeast extract peptone dextrose.

## Competing interests

The authors declare that they have no competing interests.

## Authors’ contributions

JPB initiated and coordinated the study, performed or was involved in all experiments and drafted the manuscript. RJP performed and analyzed the yeast engineering and bioconversion experiments and contributed to the manuscript draft. JMSA was involved in the design and generation of the yeast constructs and performed initial experiments. SB developed the protocols for MS detection of conversion products and participated in data generation. JED and CRS co-coordinated the study and helped in drafting the manuscript. All authors read and approved the final manuscript.

## Supplementary Material

Additional file 1: Figure S1 GAT-1 is part of the scouting machinery. Transcript abundances in fragments per kilobase of transcript per million mapped reads (FPKM) as determined by RNAseq for the *gat-1* gene (NCU00988). Sucrose pre-grown cultures were transferred to either 2% sucrose, no carbon (NoC), or 1% pectin for 4 h before tissue was harvested. Values represent means of three biological replicates (data from [37]). Clearly, *gat-1* is subject to carbon catabolite repression in presence of sucrose and is de-repressed under starvation conditions (NoC), thereby assisting in the carbon-scouting of the fungus. The gene is furthermore strongly induced by pectin. Click here for file

Additional file 2: Figure S2 Maximum likelihood phylogenetic analysis of the GAT-1 transporter. The tree was generated with the help of the phylogeny.fr software workflow [61]. In this case, the alignment was performed by MUSCLE, the phylogeny calculated by PhyML, and the tree rendered by TreeDyn (see Methods). The *S. cerevisiae* galactose transporter Gal2p was used as out-group. From the Ascomycota: A.nidulans (*Aspergillus nidulans*, Eurotiomycetes); A.niger (*Aspergillus niger*, Eurotiomycetes); B.cinerea (*Botrytis cinerea*, Leotiomycetes); F.graminearum (*Fusarium graminearum*, Sordariomycetes); M.thermophila (*Myceliophthora themophila*, Sordariomycetes); M.oryzae (*Magnapothe oryzae*, Sordariomycetes); N.crassa (*Neurospora crassa*, Sordariomycetes); P.chrysogenum (*Penicillium chrysogenum*, Eurotiomycetes); S.cerevisiae (*Saccharomyces cerevisiae*, Saccharomycotina); S.macrospora (*Sordaria macrospora*, Sordariomycetes); T.melanosporum (*Tuber melanosporum*, Pezizomycetes); T.reesei (*Trichoderma reesei*, Sordariomycetes). From the Basidiomycota: C.gattii (*Cryptococcus gattii,* Tremellomycetes); L.bicolor (*Laccaria bicolor,* Agaricomycetes); P.placenta (*Postia placenta,* Agaricomycetes); U.maydis (*Ustilago maydis*, Ustilaginomycotina); the arrow indicates the position of GAT-1 in the tree; GAT-1: XP_963898.1; quinate permease: XP_959616.1. Click here for file

Additional file 3: Figure S3 GAT-1 is not relevant for the uptake of quinic acid and GAT-1-mediated D-galacturonic acid (D-GalA) uptake is inhibited by uncouplers. (A) Cyclic and chair projections of D-GalA and D-quinic acid for comparison. (B-D) Monosaccharide transport assays. Sucrose pre-grown *N. crassa* mycelia (wild-type (WT) only in D; WT, ∆*gat-1* and ∆6026 (quinate permease) in B,C) were transferred for 4 h to 0.5% pectin (D) or 0.5% pectin + 100 μM quinic acid (*qa*) (B,C) to induce the respective response and subsequently to the reaction solution containing 90 μM each of GalA (D) or GalA + *qa* (B,C) and Vogel’s salts. The cultures were incubated in the reaction solution for 40 minutes at 25°C, 250 rpm in the light. Aliquots of the supernatant were taken at regular intervals and the remaining sugar/cyclitol concentrations analyzed by High pH anion-exchange chromatography with pulsed amperometric detection or Linear Ion Trap mass spectrometry (LTQ-MS), respectively. Bars represent standard deviations (n = 3). Click here for file

Additional file 4: Table S1 Codon optimized sequences. Click here for file

Additional file 5: Table S2 Primers used in quantitative RT-PCR experiments. Click here for file
