# Correction to: Identification and characterization of a galacturonic acid transporter from *Neurospora crassa* and its application for *Saccharomyces cerevisiae* fermentation processes

**DOI:** 10.1186/s13068-017-0955-1

**Published:** 2017-12-04

**Authors:** J. Philipp Benz, Ryan J. Protzko, Jonas M. S. Andrich, Stefan Bauer, John E. Dueber, Chris R. Somerville

**Affiliations:** 1 Energy Biosciences Institute, University of California Berkeley, Berkeley, CA USA; 2 Department of Molecular and Cell Biology, University of California Berkeley, Berkeley, CA USA; 3 Department of Bioengineering, University of California Berkeley, Berkeley, CA USA; 4 Department of Plant and Microbial Biology, University of California Berkeley, Berkeley, CA USA; 5 Institute of Environmental and Sustainable Chemistry, Technische Universität Braunschweig, Brunswick, Germany; 6 Present Address: TUM School of Life Sciences Weihenstephan, Technical University of Munich, Freising, Germany; 7 Present Address: Zymergen, Emeryville, CA USA

## Correction to: Biotechnol Biofuels (2014) 7:20 10.1186/1754-6834-7-20

The authors noticed an accidental calculation error existing in Fig. 4C of the original article [1]. The given velocities of the GAT-1 transporter (in this figure only) were mistakenly listed as 50-fold too high. Based on the correct values (Fig. 1), the new estimate of *V*_max_ for GAT-1 is 0.256 ± 0.007 nmol min^−1^ mg protein^−1^. This change will in no manner affect the conclusions/narrative of the experiments as described in the original publication. The authors apologize for any inconvenience caused.

**Fig. 1:**
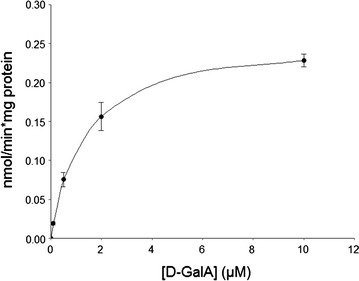
Kinetics of D-GalA transport by GAT-1 (corrected version of former Fig. 4C). The transport rate was determined as a function of D-GalA concentration (ranging from 0.1 to 10 µM) by yeast strains expressing *gat*-*1*-*sfGFP* and was normalized by total protein concentration
