# Supplementary material for: Identification and characterization of a galacturonic acid transporter from Neurospora crassa and its application for Saccharomyces cerevisiae fermentation processes
Source: Biotechnol Biofuels. 2014 Feb 6;7:20. doi: 10.1186/1754-6834-7-20 (PMC3933009; doi:10.1186/1754-6834-7-20)
Supplement: Additional file 1: Figure S1 — GAT-1 is part of the scouting machinery. Transcript abundances in fragments per kilobase of transcript per million mapped reads (FPKM) as determined by RNAseq for the gat-1 gene (NCU00988). Sucrose pre-grown cultures were transferred to either 2% sucrose, no carbon (NoC), or 1% pectin for 4 h before tissue was harvested. Values represent means of three biological replicates (data from [37]). Clearly, gat-1 is subject to carbon catabolite repression in presence of sucrose and is de-repressed under starvation conditions (NoC), thereby assisting in the carbon-scouting of the fungus. The gene is furthermore strongly induced by pectin. [file 1754-6834-7-20-S1.pdf]

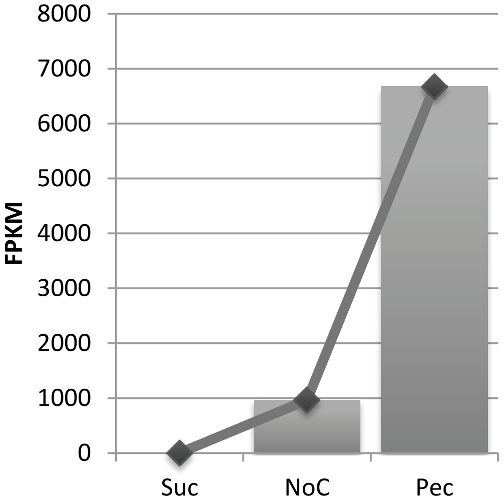

Figure S1 GAT-1 is part of the scouting machinery. Transcript abundances in FPKM as determined by RNAseq for the *gat-1* gene (NCU00988). Sucrose pre-grown cultures were transferred to either 2% sucrose, “no carbon” (NoC), or 1% pectin for 4 hours before tissue was harvested. Values represent means of 3 biological replicates (data from [37]). Clearly, *gat-1* is subject to “carbon catabolite repression” in presence of sucrose and is de-repressed under starvation conditions (NoC), thereby assisting in the “carbon scouting” of the fungus. The gene is furthermore strongly induced by pectin.
