# Supplementary material for: Identification and characterization of a galacturonic acid transporter from Neurospora crassa and its application for Saccharomyces cerevisiae fermentation processes
Source: Biotechnol Biofuels. 2014 Feb 6;7:20. doi: 10.1186/1754-6834-7-20 (PMC3933009; doi:10.1186/1754-6834-7-20)
Supplement: Additional file 2: Figure S2 — Maximum likelihood phylogenetic analysis of the GAT-1 transporter. The tree was generated with the help of the phylogeny.fr software workflow [61]. In this case, the alignment was performed by MUSCLE, the phylogeny calculated by PhyML, and the tree rendered by TreeDyn (see Methods). The S. cerevisiae galactose transporter Gal2p was used as out-group. From the Ascomycota: A.nidulans (Aspergillus nidulans, Eurotiomycetes); A.niger (Aspergillus niger, Eurotiomycetes); B.cinerea (Botrytis cinerea, Leotiomycetes); F.graminearum (Fusarium graminearum, Sordariomycetes); M.thermophila (Myceliophthora themophila, Sordariomycetes); M.oryzae (Magnapothe oryzae, Sordariomycetes); N.crassa (Neurospora crassa, Sordariomycetes); P.chrysogenum (Penicillium chrysogenum, Eurotiomycetes); S.cerevisiae (Saccharomyces cerevisiae, Saccharomycotina); S.macrospora (Sordaria macrospora, Sordariomycetes); T.melanosporum (Tuber melanosporum, Pezizomycetes); T.reesei (Trichoderma reesei, Sordariomycetes). From the Basidiomycota: C.gattii (Cryptococcus gattii, Tremellomycetes); L.bicolor (Laccaria bicolor, Agaricomycetes); P.placenta (Postia placenta, Agaricomycetes); U.maydis (Ustilago maydis, Ustilaginomycotina); the arrow indicates the position of GAT-1 in the tree; GAT-1: XP_963898.1; quinate permease: XP_959616.1. [file 1754-6834-7-20-S2.pdf]

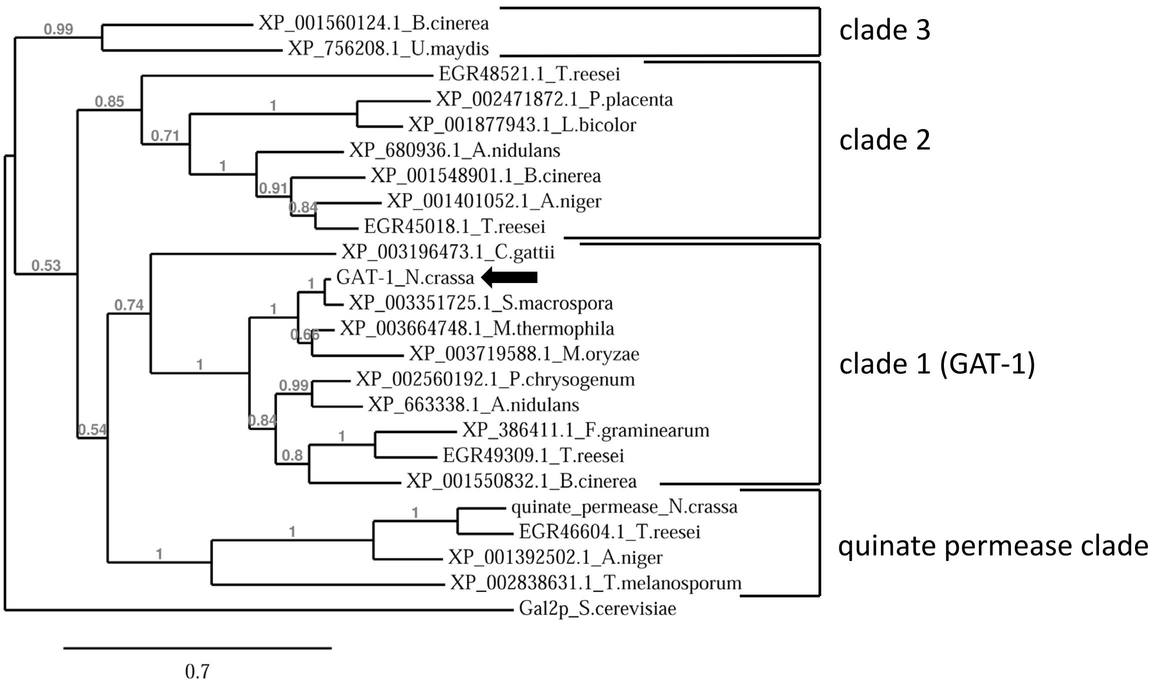

Figure S2 Maximum likelihood phylogenetic analysis of the GAT-1 transporter. The tree was generated with the help of the phylogeny.fr software workflow [60]: In this case, the alignment was performed by MUSCLE, the phylogeny calculated by PhyML, and the tree rendered by TreeDyn (see Materials & Methods). The *S. cerevisiae* galactose transporter Gal2p was used as outgroup. From the Ascomycota: A.nidulans (*Aspergillus nidulans*, Eurotiomycetes); A.niger (*Aspergillus niger*, Eurotiomycetes); B.cinerea (*Botrytis cinerea*, Leotiomycetes); F.graminearum (*Fusarium graminearum*, Sordariomycetes); M.thermophila (*Myceliophthora themophila*, Sordariomycetes); M.oryzae (*Magnapothe oryzae*, Sordariomycetes); N.crassa (*Neurospora crassa*, Sordariomycetes); P.chrysogenum (*Penicillium chrysogenum*, Eurotiomycetes); S.cerevisiae (*Saccharomyces cerevisiae*, Saccharomycotina); S.macrospora (*Sordaria macrospora*, Sordariomycetes); T.melanosporum (*Tuber melanosporum*, Pezizomycetes); T.reesei (*Trichoderma reesei*, Sordariomycetes). From the Basidiomycota: C.gattii (*Cryptococcus gattii,* Tremellomycetes); L.bicolor (*Laccaria bicolor,* Agaricomycetes); P.placenta (*Postia placenta,* Agaricomycetes); U.maydis (*Ustilago maydis*, Ustilaginomycotina); the arrow indicates the position of GAT-1 in the tree; GAT-1: XP_963898.1; quinate permease: XP_959616.1.
