# Supplementary material for: Identification and characterization of a galacturonic acid transporter from Neurospora crassa and its application for Saccharomyces cerevisiae fermentation processes
Source: Biotechnol Biofuels. 2014 Feb 6;7:20. doi: 10.1186/1754-6834-7-20 (PMC3933009; doi:10.1186/1754-6834-7-20)

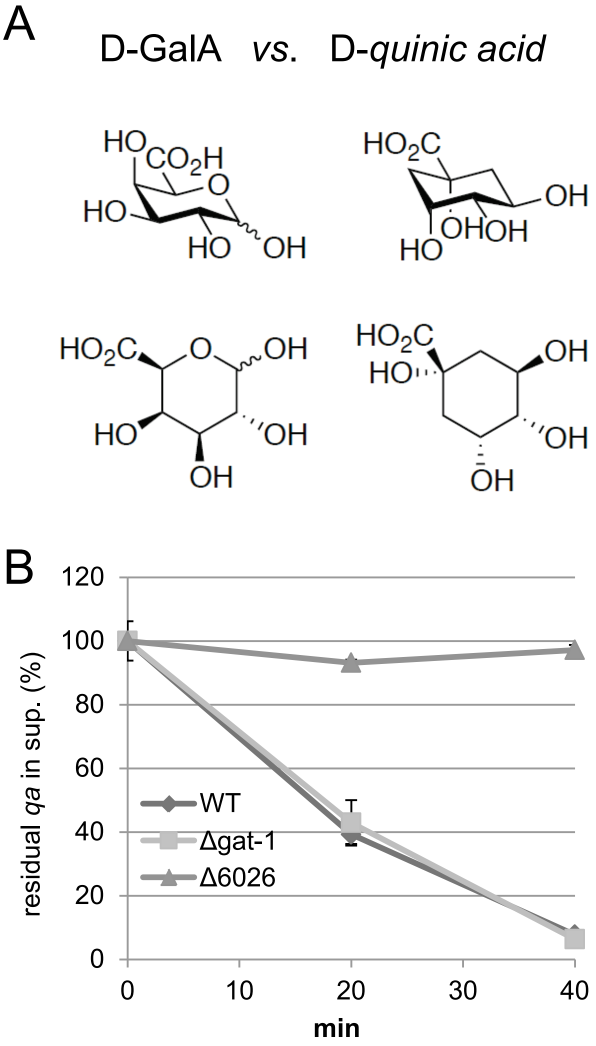

Figure S3 GAT-1 is not relevant for the uptake of quinic acid and GAT-1-mediated D-galacturonic acid (D-GalA) uptake is inhibited by uncouplers. (A) Cyclic and chair projections of D-GalA and D-quinic acid for comparison. (B-D) Monosaccharide transport assays. Sucrose pre-grown *N. crassa* mycelia (wild-type (WT) only in D; WT, Δgat-1 and Δ6026 (quinate permease) in B,C) were transferred for 4 h to 0.5% pectin (D) or 0.5% pectin + 100 µM quinic acid (*qa*) (B,C) to induce the respective response and subsequently to the reaction solution containing 90 µM each of D-GalA (D) or D-GalA + *qa* (B,C) and Vogel's salts. The cultures were incubated in the reaction solution for 40 minutes at 25°C, 250 rpm in the light. Aliquots of the supernatant were taken at regular intervals and the remaining sugar/cyclitol concentrations analyzed by 'high pH anion-exchange chromatography with pulsed amperometric detection' (HPAEC-PAD) or Linear Ion Trap mass spectrometry (LTQ-MS), respectively. Bars represent standard deviations (n = 3).

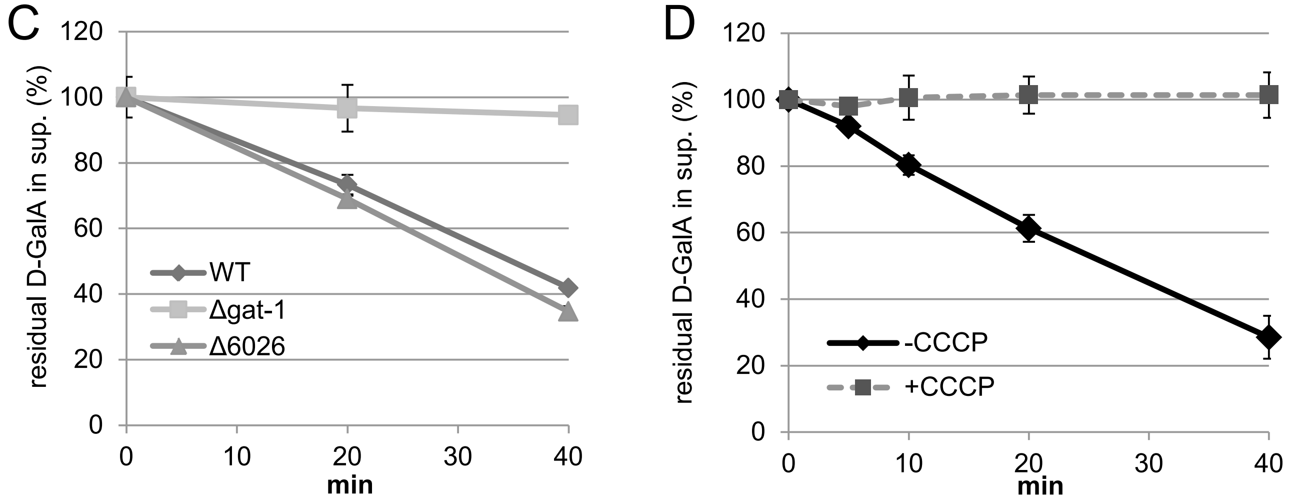

Supplement: Additional file 3: Figure S3 — GAT-1 is not relevant for the uptake of quinic acid and GAT-1-mediated D-galacturonic acid (D-GalA) uptake is inhibited by uncouplers. (A) Cyclic and chair projections of D-GalA and D-quinic acid for comparison. (B-D) Monosaccharide transport assays. Sucrose pre-grown N. crassa mycelia (wild-type (WT) only in D; WT, ∆gat-1 and ∆6026 (quinate permease) in B,C) were transferred for 4 h to 0.5% pectin (D) or 0.5% pectin + 100 μM quinic acid (qa) (B,C) to induce the respective response and subsequently to the reaction solution containing 90 μM each of GalA (D) or GalA + qa (B,C) and Vogel’s salts. The cultures were incubated in the reaction solution for 40 minutes at 25°C, 250 rpm in the light. Aliquots of the supernatant were taken at regular intervals and the remaining sugar/cyclitol concentrations analyzed by High pH anion-exchange chromatography with pulsed amperometric detection or Linear Ion Trap mass spectrometry (LTQ-MS), respectively. Bars represent standard deviations (n = 3). [file 1754-6834-7-20-S3.pdf]
