# Supplementary material for: Identification and characterization of a galacturonic acid transporter from Neurospora crassa and its application for Saccharomyces cerevisiae fermentation processes
Source: Biotechnol Biofuels. 2014 Feb 6;7:20. doi: 10.1186/1754-6834-7-20 (PMC3933009; doi:10.1186/1754-6834-7-20)
Supplement: Additional file 5: Table S2 — Primers used in quantitative RT-PCR experiments. [file 1754-6834-7-20-S5.docx]

**Table S2**

Codon optimized sequences.

*Neurospora crassa* galacturonic acid transporter-1 (GAT-1) with C-terminal superfolder green fluorescent protein (underlined) codon optimized sequence for yeast expression

atgggtctttcgataggaaataggatcctccggaaaattgtcaaaaatgaggccatggcagaagatcccccagagatctatggctggcgtgtctatctcctagcgtgctctgcctgcttcggcgccatgtctttcggctgggattcctccgtcatcggcggcgtcatcgaactcgaaccctttaaacacgactttggcttcatcggcaacgataaagccaaggccaacctgggcgccaatatcgtctctaccctccaagccggctgcttcctcggtgcgctgatcgcctcacctataaccgatcgcttcggccgcaagtggtgtctcatcgctgtctccctggtcgtcatcatcggtatcatcatgcaagccgccgcctcaggcaacctcgcacccatgtacattggccgtttcgtcgccggcgtgggcgtcggcgccgccagctgcatcaaccccgtctttgtgtctgagaacgctccccgctcgatccgcggtctgttgacgggcctctaccaactcttcattgtcaccggcggcatgatcgcattctggatcaactactccgtctctctgcacttcaagggcaaatccatgtacatcttcccgctcgccatccaaggtcttcccgccggccttttgtgcgtctgcatgctcctctgccacgaaagcccgcgctggctggcccgtcgtgaccgatgggaagaatgcaagtctgtgctggcgcgcatccgcaacctccccccagaccacccgtacatcgtcgacgagttccgcgagatccaggaccagctcgaacaggagcgtcgtctccagggcgacgccacttactgggacttgacccgcgatatgtggaccgtcgccggcaaccgcaagcgcgccctgattagtattttcttgatgatctgccagcaaatgacgggcaccaacgccatcaacacgtacgcgcctaccatcttcaagaacttgggtatcaccggcacgtcgactagcttgtttagtaccggcatctatggtattgtcaaggtcgttagctgcgtcattttcttgctgttcttggccgactcgctgggtcgtagacgttcgctgctgtggacgtcgattgcgcagggtcttgctatgttttatattggcctttatgtccgcatctcgccgccgattgatggccagccggtgccgcctgcgggttatgtagcgttggtgtgcatatttctgtttgctgctttcttccaatttggctggggtcctgcctgctggatctacgcctcggaaatccccgccgcccgcctgcgctccctcaacgtgtcctacgccgccgcgacgcagtggctgttcaatttcgtcgtggcccgcgccgtgcctactatgctggtcacggtcggcccccacggttacggcacctacctcatctttggcagcttctgcctcagcatgtttgtctttgtctggttcttcgtgcccgagacaaagggtatctcgcttgagcacatggatgagctgtttggcgttactgatgggcctgccgctgagaagtcgtcggtgcatggtggagatgatgtcgggtcggagatggggaagggggatcagaagtcgaagcatgtggaggtttatgttatcgatggtagtggtagtgtgagcaagggcgaggagctgttcaccggggtggtgcccatcctggtcgagctggacggcgacgtaaacggccacaagttcagcgtgcgcggcgagggcgagggcgatgccaccaacggcaagctgaccctgaagttcatctgcaccaccggcaagctgcccgtgccctggcccaccctcgtgaccaccctgacctacggcgtgcagtgcttcagccgctaccccgaccacatgaagcagcacgacttcttcaagtccgccatgcccgaaggctacgtccaggagcgcaccatcttcttcaaggacgacggcacctacaagacccgcgccgaggtgaagttcgagggcgacaccctggtgaaccgcatcgagctgaagggcatcgacttcaaggaggacggcaacatcctggggcacaagctggagtacaacttcaacagccacaacgtctatatcatggccgacaagcagaagaacggcatcaaggtgaacttcaagatccgccacaacgtcgaggacggcagcgtgcagctcgccgaccactaccagcagaacacccccatcggcgacggccccgtgctgctgcccgacaaccactacctgagcacccagtccgtcctgagcaaagaccccaacgagaagcgcgatcacatggtcctgctggagttcgtgaccgccgccgggatcactctcggcatggacgagctgtacaagtag

*Aspergillus niger* d-galacturonic acid reductase (GAAA) codon optimized sequence for yeast expression

atggcccctcccgccgttttaatggtaggaaccggtgaatataccacaggttacgttggtggaactgcatcaacttctgataaaaaggtaggtgtagttgggctcaccctctttgatctgcgaagaagaggtaaagtgggtgacttatctatggtgggagtttccggcagtaaatttccaggcatacgcgcacatcttcaaaaaaatatctcggaagtatacaatggtcttgatgtgtcttttacttctttcccggctgataatactagcgatcccgaggcttataaggctgctattgatgctctccctgcgggtagcgctataaccattttcacgccagatcctacacattatcccatcgccttatatgcgatacaaaggaagattcatgtactaattactaagccggccactaagctattgtccgatcatttagacttacttgctgaatcaagaaaacataatgtggtggtttatattgagcaccacaaaaggtttgatcctgcctattctgatgctcgtgctaaggcagcaaaattaggcgactttaattatttttactcttatatgtcccaacctaaaagtcaactggagacttttaaagcttgggcaggcaaggatagcgatatctcctattatctaaattcccatcatgttgacgttaacgaatcgatggttcccgattatgtgccggtgaaggtcaccgctagtgcagctacgggcacagcagtggaattggggtgtgcacacgaaacggaagatactattactctattggtggaatggaagaaaaaggatgggtcaagaatggccactggcgtctacacatcttcgtggaccgcacctcagagagctggtgtccactcgaatcagtattttcactatatgggttcgaaaggggagatcagagttaaccaagcgaaaaggggttatgacgttgcagaggatgaggccggcttgtcttggattaaccctttttatatgaagtatgctcctgatgaagaaggcaattttggaggtcagactggatacggttatatttcttttgaaaaattcatagacgcagttacagccgttaatgaagggagattgactctggatcaactggacgcgcgtccaattcccactttaaagaacactatcgcgacaactgcaattttacacgctggcaggatcagcttggatgaaaaaaggtcggttgagattgttacagaagatggtaaatgggagttaaaatag
